# Clinical aspects of a large group of adults with Angelman syndrome

**DOI:** 10.1002/ajmg.a.61940

**Published:** 2020-10-27

**Authors:** Inge den Besten, Rianne F. de Jong, Amber Geerts‐Haages, Hennie T. Bruggenwirth, Marije Koopmans, ENCORE Expertise Center for AS 18+, Alice Brooks, Ype Elgersma, Dederieke A. M. Festen, Marlies J. Valstar

**Affiliations:** ^1^ Intellectual Disability Medicine, Department of General Practice Erasmus MC Rotterdam The Netherlands; ^2^ Department of Clinical Genetics Erasmus University Medical Center Rotterdam The Netherlands; ^3^ Department of Medical Genetics Utrecht University Medical Center Utrecht The Netherlands; ^4^ Department of Neuroscience Erasmus MC University Medical Center Rotterdam The Netherlands; ^5^ ASVZ, Medical Department Care and Service Centre for People with Intellectual Disabilities Sliedrecht The Netherlands

**Keywords:** Angelman syndrome, clinical spectrum, intellectual disability, natural history, UBE3A

## Abstract

Descriptions of the clinical features of Angelman syndrome (AS) have mainly been focused on children. Here, we describe the evolution of the clinical phenotypes of AS in adulthood, using clinical data from 95 individuals (mean age 31.6 years, median 29.0 years, range 18–83 years), with genetically confirmed AS. Data was collected through physical examination and inspection of medical records, combined with questionnaires and interviews. Adults with AS experience substantial debilitating health problems. Constipation, reflux, visual problems, scoliosis, behavioral and sleeping problems occurred frequently and require appropriate attention. Epilepsy was reported in 57% of adults, negatively affecting the level of functioning. Non‐convulsive status epilepticus was not observed in the adults, however some individuals developed prolonged episodes of rhythmic shaking while awake. A decline in mobility was noted in the majority of adults. A minority of adults with AS showed microcephaly. Taken together, this first phenotypic study of adults with AS to include in person interviews with care‐givers and physical examination of patients, including the eldest adult reported to date, provides important insight in the development of the syndrome into adulthood. This knowledge is required to improve care for adult individuals with AS and to evaluate future therapies for this group.

## 1 INTRODUCTION

Angelman syndrome (AS) is a rare neurogenetic disorder with an estimated prevalence of about 1:20.000 (Mertz et al., 2013). AS is caused by loss or dysfunction of the maternal *UBE3A* gene, which is in the brain exclusively expressed from the maternally inherited chromosome 15. There are four different genetic or epigenetic causes that can lead to AS. An interstitial deletion of the 15q11‐13 region of the maternally inherited chromosome is the most common cause of AS in approximately 70% of the individuals with AS. Other genetic causes are uniparental disomy (UPD), imprinting center (IC) defects and mutations in the *UBE3A* gene itself (Buiting, Williams, & Horsthemke, 2016).

AS is clinically characterized by severe cognitive impairment, epilepsy, poor sleep, motor problems ataxia, and speech impairment. Individuals with AS have a typical behavioral phenotype that includes interest in social interaction, frequent laughing, and hyperactivity. In addition they often have facial dysmorphisms, such as microcephaly, brachycephaly, wide spaced teeth, pointed chin, and macrostomia. Clinical criteria of AS have been described by Williams and colleagues (Williams et al., 1995; Williams et al., 2006).

Since Harry Angelman described AS for the first time in 1965 (Angelman, 1965), many studies have reported on AS in childhood (Bindels‐de Heus et al., 2020; Mertz et al., 2014; Tan et al., 2011; Thibert, Larson, Hsieh, & Raby, 2013). This resulted in a clear picture of the health issues of children with AS. Far less is known is about the course of the syndrome in adulthood. Only a limited number of studies described the course of the AS in adults (Buckley, Dinno, & Weber, 1998; Clayton‐Smith, 1993; Giroud et al., 2015; Guerrini et al., 1996; Jacobsen et al., 1998; L. A. E. M. Laan, Den Boer, Hennekam, Renier, & Brouwer, 1996; Leitner & Smith, 1996; Minassian et al., 1998; Moncla et al., 1999; Penner, Johnston, Faircloth, Irish, & Williams, 1993; Sandanam et al., 1997; A. Smith et al., 1996; Williams & Frias, 1982; Williams, Gray, Hendrickson, Stone, & Cantu, 1989). These studies mostly reported on small numbers of patients, the largest study describes 28 patients (J. C. Smith, 2001). One recent study described a cohort of 110 adults (Larson, Shinnick, Shaaya, Thiele, & Thibert, 2015). This study was based on telephone interviews with parents or guardians. The adults were not seen and a study of medical records was not included. In addition, the mean age of this cohort is only 24 years. Adults with AS over 40 years are rarely described in the literature, even though adults with AS are anticipated to have normal life expectancy, based on sporadic case reports of adults with AS over 50 (Bjerre, Fagher, Ryding, & Rosen, 1984; Buckley et al., 1998; Duker, Van Driel, & Van De Bercken, 2002; Jacobsen et al., 1998; L. A. E. M. Laan et al., 1996; Meijers‐Heijboer et al., 1992; Ronan, Buiting, & Dudding, 2008), however good data about life expectancy is still missing.

The aim of this study is to describe the evolution of AS in adulthood. By gaining more knowledge of the clinical spectrum of AS in adulthood, care can be optimized to improve quality of life for adults with AS. At the same time, natural history data will serve as an essential reference to evaluate future therapies that will be used in the adult population.

## 2 METHODS

From 2015 to 2018 adults with genetically confirmed AS were included in this study to investigate the natural course of AS in adulthood. Adult individuals with AS were recruited for the study via the Dutch Angelman parents association (vASN; Vereniging Angelman Syndroom Nederland) and the Dutch Society of Physicians for persons with intellectual disabilities. Furthermore adults visiting the outpatient clinic of the Dutch *ENCORE* expertise center for neurodevelopmental disorders (which includes the national AS expertise center) were approached to participate.

Since adults with AS have an expressive language deficit and severe cognitive impairment, they were considered incapable of giving informed consent. Therefore, legal representatives, usually court appointed parents or family members, who were interested in participation of their ward in the study, were sent information regarding the study to decide upon participation of their ward in the study. Written informed consent of the legal guardian was thereafter obtained.

The study was approved by the Medical Ethical Review Board of the Erasmus Medical Center, Rotterdam (MEC‐2015‐267). Written informed consent was obtained for all participants.

Three questionnaires were filled in by parents and/or caregivers of participants. First a questionnaire requesting information on pregnancy, first clinical signs and symptoms, mental and motor milestones, behavioral problems, sleeping problems, epilepsy, hearing and vision, digestion and medical history. Secondly, two standardized questionnaires were used: the Aberrant Behavior Checklist‐community (ABC‐C) and Sleep Disturbance Scale for Children (SDSC) (Bruni et al., 1996; Marshburn & Aman, 1992).

The ABC‐C is a behavior scale developed by Aman et al. to measure treatment effectiveness for people with intellectual disabilities (Aman, Singh, Stewart, & Field, 1985). It consists of 58 items, which indicates five subscales: irritability, lethargy‐social withdrawal, stereotypic behavior, hyperactivity and inappropriate speech. Parents or caregivers can rate each item on a four‐point scale ranging from 0 (not a problem) to 3 (severe problem). The ABC‐C has shown high internal consistency, good inter‐rater reliability, and a consistent five‐factor structure in multiple studies (Brinkley et al., 2007; Brown, Aman, & Havercamp, 2002). Since the subscale “inappropriate speech” was irrelevant to the AS population given that the vast majority of individuals are nonverbal, we omitted the inappropriate speech scale in this study.

The Sleep Disturbance Scale for Children (SDSC) is a screening test to identify children with disturbed sleep. The SDSC is a 27‐item inventory rated on a 5 point Likert‐type scale. The instrument's purpose is to categorize sleep disorders in children. As well as giving an overall score the instrument uses five subdomains: disorders of initiating and maintaining sleep, sleep breathing disorders, disorders of arousal, sleep–wake transition disorders, disorders of excessive somnolence, and sleep hyperhidrosis. According to the developers of the test, the SDSC had good diagnostic accuracy (AUC = 0.9 1) (Bruni et al., 1996).

During an interview with parents and/or caregivers the completed questionnaires were discussed and corroborated. All questions were asked in the same manner, following the questionnaires outline and data were interpreted by the researchers together (IdB, AGH, MJV). Clinical data were complemented by studying the medical records.

In addition, parents and/or caregivers were interviewed with the Vineland Adaptive Behavior Scales (VABS) by one of the researchers (IdB, RFdJ, AGH, MJV). The VABS is a widely used instrument to obtain an measure of adaptive functioning on the domains communication, daily living skills, socialization and motor skills indicating an estimated developmental age (Sparrow & Cicchetti, 1985). The reliability of the VABS in children and adolescents with intellectual disabilities is proved to be good and the construct validity is high (de Bildt, Kraijer, Sytema, & Minderaa, 2005).

All adults were seen for physical examination, which took place immediately after the interview.

During physical examination, the focus was on the general health condition, growth, skin, throat, nose and ears, eyes, lymph nodes, heart, lungs, abdomen, back, arms, legs and joints. Photos were taken of each adult and they were recorded on video, in order to describe the facial characteristics and gait, if participants were ambulatory. To reduce the burden of participation in the study for the adults with AS and their caregivers, they were visited in their home environment by the researchers.

The study was mainly cross sectional. Physical examination, VABS and SDSC were performed once. Through the general questionnaire, interviews and the study of medical records, a retrospective overview of the medical history and clinical course was assembled.

For all participants the results of genetic tests were checked. The diagnosis of AS had been confirmed by the standard tests at the time of diagnosis, including methylation sensitive digestion, methylation‐specific PCR (MS‐PCR), methylation‐specific multiplex ligation‐dependent probe amplification (MS‐MLPA), microsatellite marker analysis (MSA), fluorescent in situ hybridization (FISH), MLPA analysis of copy number, Comparative Genomic Hybridization (CGH)‐ or Single Nucleotide Polymorphism (SNP)‐array or single *UBE3A* gene sequencing (Beygo et al., 2019).

Data were analyzed using SPSS version 25 for windows. Descriptive statistics and frequencies were used for calculations. For genotype–phenotype correlations, adults with pathogenic variants in UBE3A and UPD were grouped into a “non‐deletion” group, and compared to adults with a deletion of the 15q‐11q13 PW/AS region (Beygo et al., 2019). Differences between the groups were calculated with independent *t*‐tests for normally distributed continuous data or categorical data a *χ*2 test was performed. For non‐normally distributed data Mann Whitney *U* test was used.

All statistical tests were two‐sided and *p*‐values <.05 were considered statistically significant.

## 3 RESULTS

### 3.1 Patients

Hundred and one adults with genetically confirmed AS were initially included in the study. Six individuals from one large family, with a novel maternally‐inherited *UBE3A* sequence variant, presented with a phenotype that did not match the clinical criteria for AS. These individuals were excluded from this report and were described separately (Geerts‐Haages et al., 2020).

The remaining 95 adults (48 males, 47 females) were included in this study (Table 1). For 11 of these adults childhood data were included in the Dutch study on health issues and development in children with AS (Bindels‐de Heus et al., 2020). The age ranges from 18 to 83 years, with a mean age of 31.6 years (median 29.0 years, range 18–83 years). Type of genetic cause and type of genetic test was recorded. In 19% of the cases the confirmed diagnosis was based on an aberrant methylation test and no additional tests were done to specify the subtype, they were classified as “undefined” (Table 1). Except for two twin brothers with a pathogenic variant in UBE3A, none of the adults were related.

**TABLE 1:** Patient characteristics

	Full cohort (*n* = 95)	%
Mean age	31.6 (*SD*: 12.6; range: 18–83)	
Median age	29.0 (*SD*: 12.6; range: 18–83)	
18–29 years	48	50.5
30–39 years	25	26.3
40–49 years	15	15.8
50–59 years	3	3.2
>60 years	4	4.2
Sex		
Male	48	50.5
Female	47	49.5
Ethnicity^a^		
Dutch	85.5	90.0
Belgian	2	2.1
Moroccan	2	2.1
Surinamese	2	2.1
Turkish	1	1.1
American	1	1.1
German	0.5	0.5
Georgian	0.5	0.5
Spanish	0.5	0.5
Genotype		
Deletion	56	58.9
UBE3A pathogenic variant	15	15.8
UPD	6	6.3
Undefined^b^	18	18.9

Abbreviation: UPD uniparental disomy.

^a^ The ethnicity of parents was considered to describe ethnicity of the adults, with parents together counting for 1. So if, for example, father came from Spain and mother from the Netherlands, we noted 0.5 for both groups.

^b^ All patients have genetically confirmed AS, but in some cases there were no additional tests results available (only DNA methylation test). Therefore, no distinction can be made between a deletion and UPD in these cases.

Ninety‐three adults were seen at least once over a period of more than 3 years, from May 2015 till February 2019. For the two remaining adults, permission was given to study medical records and send questionnaires only. Table 2 shows an overview of the number of available information sources. For some adults, parents and/or caregivers were visited and adults were seen, but no permission was given for physical examination.

**TABLE 2:** Sources of information

	Full cohort (*n* = 95)	%
Physical examination	86	90.5
Natural course questionnaire	93	97.9
Aberrant behavior checklist (ABC‐C)	89	93.7
Sleep disturbance scale for children (SDSC)	88	92.6
Vineland adaptive behavior scales (VABS)	92	96.8
Medical records	88	92.6

### 3.2 Pregnancy and birth

Data on pregnancy was available for 91 cases. 77% of the pregnancies were uneventful. Four cases of pre‐eclampsia or hypertension were reported and six cases of vaginal bleeding during pregnancy. The remaining cases mentioned hyperemesis gravidarum, pyelonephritis or frequent cystitis, hypothyroidism, extreme tiredness or limited movement of the fetus.

The duration of the pregnancy was known in 87 cases. There was a term pregnancy in 91%. The remaining 9% was late pre‐term and one individual was born preterm at 32 weeks. The birth weight was known in 80 cases. The mean birth weight of a term individual with AS was 3,117 g (range 1950–6,000). This is slightly lower than the mean birth weight of Dutch newborns, which is 3,431 g (reference CBS 2011/2013).

Six twin pregnancies were identified in 94 pregnancies. In these twin pregnancies, intra uterine fetal death occurred twice. Three twins consisted of one baby with AS and one unaffected baby, and in one twin pair both children were affected with AS as a result of and UBE3A variant. The average maternal age during these twin pregnancies was 32 years (range 26–39 years).

In the majority (82%) of adults, the first signs / problems started in the first few months after birth. In 65% of these individuals, feeding problems were reported, such as little or no sucking reflex, poor swallow and frequent vomiting. In other individuals, poor sleeping, excessive crying, icterus, heavy sweating, hernia inguinalis, postnatal hypothermia, arching, absence of primitive reflexes such as walking reflex and palmar grasp reflex, anal fistula, and anusatresia were mentioned. Although the first symptoms of AS started early, the median age of diagnosis in this cohort is 6.8 years (mean 12.8 years, range 9 months–65.2 years). Notably, the age of diagnosis is lower in younger individuals, probably reflecting improved availability of genetic testing. The median age of diagnosis in the age group of 18–29 years is much lower with 2.5 years (mean 3.6 years, range 9 months–14 years).

### 3.3 Behavior

Behavioral data was available for 93 adults. In 86%, parents and/or caregivers mentioned inappropriate behavior of the participant. Hair pulling was mentioned most often, followed by hitting, screaming, skin and nail picking, pinching, and biting. In more than 25%, this behavior was considered problematic. However, the majority of parents and/or caregivers indicated that this behavior was justifiable and perceived this behavior rather as inappropriate communication rather than a behavioral problem. Sixteen percent of the group with behavioral problems used medication, most often Risperidone.

While hyperactivitity occurred in childhood in most individuals, the majority of adults have become much calmer over the years, according to their parents and/or caregivers. This decrease in hyperactivity was already noticed in the youngest age group of 18–30 years. ABC‐C subscale data are presented in Table 3.

**TABLE 3:** SDSC and ABC‐C subscales

SDSC	DIMS	SBD	DA	SWTD	DOES	SHY
(*n* = 69)	[7–35]^a^	[3–15]^a^	[3–15]^a^	[6–30]^a^	[5–25]^a^	[2–10]^a^
Mean (*SD*)	14,09 (5,28)	4,16 (1,81)	3,06 (0,34)	7,78 (2,73)	8,61 (3,73)	2,62 (1,44)
Median	13,00	3,00	3,00	6,00	7,00	2,00
Range	7–21	3–12	2–5	5–18	5–21	2–10

ABC‐C (*n* = 87)	Irritability [0–45]^a^	Lethargy [0–48]^a^	Stereotypy [0–21]^a^	Hyperactivity [0–48]^a^
Mean	7.25	4.52	2,62	8,92
(SD)	(7.38)	(5,48)	(3,53)	(8,1)
Median	6	3	1	8
Range	0–33	0–26	0–15	0–39

Abbreviations: ABC‐C, Aberrant Behavior Checklist‐Community; DA, arousal disorders; DIMS, difficulty in initiating and maintaining sleep; DOES, disorders of excessive somnolence; SBD, sleep breathing disorders; SDSC, Sleep Disturbance Scale for Children; SHY, sleep hydrosis; SWTD, sleep–wake transition disorders.

^a^ Theoretical range of subscale scores.

### 3.4 Epilepsy

Eighty‐four adults had a history of seizures (Table 4). Of these, 45% were seizure‐free for at least 1 year, the majority with the use of antiepileptic drugs (AED). Medication failed to control epilepsy in 11%. This group of adults suffered at least monthly from tonic–clonic (TC) seizures or had daily smaller seizures, mostly atypical absences. We defined this group as the “no control/active seizures”‐group. The remaining 44% of adults had partial control of their epilepsy, most of them had absences.

**TABLE 4:** Epilepsy parameters

	Cohort (*n* = 94)	%
History of seizures	84	89.4
Current seizures	*n* = 84	
Seizure free/off AEDs	9	10.7
Seizure free/AED Tx	29	34.5
Partial control	37	44.0
No control/active seizures	9	10.7

Abbreviation: AED, antiepileptic drug.

Episodes of non‐convulsive status epilepticus had occurred in some participants in childhood, with recurrent hospitalization reported in five individuals. These episodes were not reported in adulthood. In a number of adults prolonged episodes of rhythmic shaking without loss of consciousness occurred. These episodes could be triggered by menstruation, tiredness of sudden changes of position.

The average age at onset of epilepsy was 3.3 years. Seventy‐two percent had their first insult before the age of 5 years and 22% were between 5 and 10 years old during their first insult. Of the remaining five individuals, a first insult occurred at 10, 12, 13, 20 or 22 years of age. We found no correlation between the degree of response to AEDs and the age at onset.

Out of 84 adults who had a history of seizures, 80% used AEDs at the time of study. Most adults used one or two medicines in an attempt to control the epilepsy. Valproate was the most commonly used (49 times), followed by clobazam (16), levetiracetam (10), clonazepam (9), ethosuximide (8), lamotrigine (8), carbamazepine (7), fenobarbital (2), and nitrazepam (1).

The severity of epilepsy and the VABS data are presented in Table 5. We found no clear correlation between the current degree of seizure control and developmental age calculated by the VABS questionnaire. Participants without epilepsy did however have significant higher VABS scores on the domains socialization and daily living skills compared to participants with epilepsy. On the domain motor skills participants without epilepsy achieved higher scores, although not significant. The communication scores were not significantly different between the groups.

**TABLE 5:** Degree of epilepsy versus mean developmental age in months

	Cohort (*n* = 92)	Range in full cohort	No history of seizures (*n* = 10)	Seizure free (*n* = 37)	Partial control (*n* = 36)	No control (*n* = 9)
Communication	12	4–30	15	12	13	10
Daily living skills	19	6–59	30	17	19	16
Socialization	12	2–52	17	11	12	12
Motor skills	18	1–37	25	17	18	14

### 3.5 Sleep problems

Sleep data is presented in Table 6. Eighty‐one adults had a history of sleep problems, according to their parents and/or caregivers. Difficulty falling asleep, frequent waking and waking up too early were mentioned most. For 25% of individuals with a history of sleep problems, no change had occurred in the degree of sleep problems. In the vast majority of the adults, an improvement or even disappearance of the problems had been mentioned as they became older. Forty‐one percent of the adults with a history of sleep problems used sleep medication, mainly melatonin with variable doses (range 0.1–5 mg). We did not find relations between sleep problems, severity of epilepsy and behavioral problems.

**TABLE 6:** Sleep parameters

	Cohort (*n* = 92)	%
History of sleep problems	81	88.0
Course of sleep problems	*n* = 81	
Disappeared with age	29	35.8
Improved with age	30	37.0
Unchanged	20	24.7
Worsened with age	2	2.5
Sleep medication	33	40.7
Melatonine	28	34.6
Other sleep medication^a^	6	7.4

^a^ Promethazine, Alimemazine, Metroprolol, Midazolam, Clonazepam, Clobazam.

Data from the Sleep Disturbance Scale for Children (SDSC) was available for 88 adults, but due to missing values a score could be calculated for 69 adults only. Nineteen percent of the adults had a pathologic score, 1% a borderline score and 12% scored suspect for a sleeping disorder. Sixty‐eight percent had a normal score. In total 32% had behavioral sleep disturbance concerning for a formal sleeping disorder according to the SDSC. Parents and/or caregivers reported sleep problems in 57% of the adults at time of the study.

### 3.6 Vision and hearing

Data about visual functioning were available for 92 adults. Of these adults, 67% had a good vision, according to their parents and/or medical records. For 30% of adults, a reduced vision had been mentioned, but only 18% wore glasses. Strabismus was seen in 32%, of which 2 adults had surgery in an attempt for correction. Two older individuals of 63 and 83 years old suffered from cataracts, for one of them the cataract had been treated with surgery. The decreased vision in this adult was noted after she started to fall more frequently. In the postoperative period, this was fully resolved.

Hearing was reported sufficient in 88 out of 90 adults, although proper testing was rarely performed.

### 3.7 Mobility

The majority of adults had been able to walk, with or without support at some point in life, as shown in Table 7. The mean age at which these adults started walking was 4.1 years. Only three adults had never been able to walk. Thirty percent of the adults had been able to walk long distances, which was defined as walks of several kilometers and/or being able to walk during days out/trips. Another 39% did walk independently, but only over short distances (10–100 m or >100 m).

**TABLE 7:** Degree of mobility

	Cohort *n* (%)	18–29 years	30–39 years	40–49 years	50–59 years	>60 years
Mobility in the home	*n* = 91	*n* = 45	*n* = 24	*n* = 15	*n* = 3	*n* = 4
Walks independently	59 (65)	30	15	11	2	1
Side arm assist or walking frame	12 (13)	3	5	3	—	1
Non‐ambulatory	20 (22)	12	4	1	1	2
Mobility outside the home	*n* = 89	*n* = 45	*n* = 23	*n* = 14	*n* = 3	*n* = 4
Walking independently	47 (53)	26	13	7	1	—
Side arm assist or walking frame	8 (9)	1	3	3	—	1
Wheelchair	34 (38)	18	7	4	2	3
Maximum distance at some point in life	*n* = 92	*n* = 47	*n* = 24	*n* = 15	*n* = 3	*n* = 3
Never been able to walk	3 (3)	3	—	—	—	—
Walks with support	25 (27)	13	8	1	1	2
Walks 10–100 m	12 (13)	6	3	2	1	—
Walks >100 m	24 (26)	7	8	8	—	1
Long distances	28 (30)	18	5	4	1	—
Mobility compared to the past	*n* = 86	*n* = 43	*n* = 24	*n* = 12	*n* = 3	*n* = 4
Unchanged	42 (48)	29	10	1	1	1
Mild decline	23 (27)	8	8	6	1	—
Serious decline	19 (22)	5	5	5	1	3
Improved	2 (2)	1	1	—	—	—

Abbreviation: BMI, body mass index.

At the time of study, a mild decline in mobility was noticed in 27% of the adults, which was defined as more rigid or uncertain walking, more in need of support and/or more difficulty in climbing the stairs. Twenty‐two percent showed a serious decline, which was defined as a shortening of the maximum walking distance, falling more frequently or switching from walking into crawling. In 84% of the adults 40 years and older, a decline in mobility was reported. Of the adults who had been able to walk long distances, none showed a serious decline in mobility. Fifteen adults showed no change at all and for nine individuals only a mild decline was reported. These nine adults were scattered across all age categories. Decline in mobility is already noted in some individuals from puberty, but is seen and is increasing in every age category. In adults with visual problems, 61% showed a decline in mobility, compared to 42% of the adults without visual problems. However, this difference was not statistically significant.

### 3.8 Nutritional status

Data was available for 91 adults. Almost half of the adults had trouble to master themselves when it came to food. They could not stop eating, were insatiable or ate too greedy. For 12% of these adults, the parents and/or caregivers mentioned that the adult is nowadays more in control. Ten percent of the adults were described as poor eaters.

Concerning constipation, data was available for 92 adults. Almost 90% suffered from constipation. Of these 65% used polyethylene glycol with the aim to control the constipation.

Dysphagia or frequent choking was reported in 45% of participants at time of study. Adults with dysphagia or frequent chocking had pneumonia in medical history twice as often compared to adults with no known swallowing disorder, respectively 32% and 15%. Approximately one third of the adults suffered from gastroesophageal reflux.

### 3.9 Physical examination

Anthropometric and orthopedic data are presented in Table 8. The average length of the males was 173 cm and of the females this was 162 cm. Twenty‐five percent of the women were at least two standard deviations below the mean length of the Dutch population. In men, this was the case for 22% of individuals.

**TABLE 8:** Anthropometrics and orthopedics

	Total cohort	%	Male	Female
Length	*n* = 79		*n* = 36	*n* = 43
Mean	167 cm (*SD*: 9.94)		173 cm (*SD*: 7.74)	162 cm (*SD*: 8.80)
Normal	60	75.9	28	32
Small in length (≤ −2 *SD*)	19	24.1	8	11
Weight	*n* = 78		*n* = 37	*n* = 41
Mean	68 kg (*SD*: 13.91)		69 kg (*SD*: 13.19)	67 kg (*SD*: 14.64)
BMI 18.5–25	45	57.7	24	21
Underweight (BMI < 18,5)	4	5.1	4	—
Overweight (BMI ≥25)	20	25.6	7	13
Obese (BMI ≥30)	9	11.5	2	7
Scoliosis	*n* = 94		*n* = 47	*n* = 47
None	44	46.8	24	20
Mild	29	30.9	12	17
Severe	11	11.7	7	4
Surgical intervention	7	7.4	3	4
Corset treatment	3	3.2	1	2
Postural abnormalities	*n* = 77		*n* = 32	*n* = 44
Pes planus	29	37.7	16	13
Pes equinus	8	10.4	5	3
Pes cavus	1	1.3	—	1
Other foot abnormalities	7	9.1	—	6
Other contractures	18	23.4	12	5

Weight data was available for 78 adults. The mean Body Mass Index was 24. One in ten men was underweight (BMI <18,5). There was no underweight in the female group. In total almost 60% had a healthy weight. Thirty‐seven percent was overweight (BMI ≥25), of which one third was obese (BMI ≥30). The male–female ratio in the group with overweight/obesity was not quite equal. Among the men, 24% was overweight and in the female group almost 50% was overweight.

Fifty percent of the adults showed a foot abnormality and contractures existed in 22% of the adults, knees and hips contractures were most frequent.

The majority of the adults had scoliosis. Of these 64% suffered from mild scoliosis, of which 9% had worn a brace during puberty. In 22% of individuals, severe scoliosis was observed, of which more than 80% never had any form of therapy. One adult developed a severe scoliosis despite wearing a brace and another one had worn a brace and had undergone surgery, but still developed a severe scoliosis. Fourteen percent have undergone surgery to correct their scoliosis successfully. Five of the 11 adults with severe scoliosis were able to walk without aid, the remaining 54% (6/11) were wheelchair bound. Of the adults with mild scoliosis or with brace or surgical corrected scoliosis, approximately 60% were able to walk independently and 16% with side arm assist/walking frame. Twenty‐five percent were wheelchair bound. In the group without scoliosis, only 11% were wheelchair‐bound.

Facial features are presented in Table 9.

**TABLE 9:** Facial characteristics

	Cohort size	Characteristic present	%
Flat occiput	91	65	71.4
Pointed chin	92	50	54.3
Prominent lower lip	92	49	53.2
Dribbling	92	46	50.0
Macrostomia	91	43	47.3
Microcephaly	88	27	30.7
Strabismus	91	30	33.0
Wide spaded teeth	82	18	22.0

### 3.10 Development and skills

Average estimated developmental ages, as measured with the VABS are presented in Table 10. As the table shows, there is little difference in mean developmental age when it comes to adults from 18 to 50 years old. The group aged 50 to 59 showed a remarkably higher average development age, but this small group consisted of two adults with a UBE3A variant and one adult with an undefined AS genotype. Adults older than 60 years scored lowest.

**TABLE 10:** Mean developmental age in months according to Vineland Adaptive Behavior Scales (VABS)

	Full cohort (*n* = 92)	Range in full cohort	18–29 years (*n* = 47)	30–39 years (*n* = 24)	40–49 years (*n* = 14)	50–59 years (*n* = 3)	>60 years (*n* = 4)
Communication	12.5	4–30	12.6	12.0	12.9	20.3	7.0
Daily living skills	19.0	6–59	18.8	17.6	21.4	35.0	9.3
Socialization	12.3	2–52	12.4	11.3	11.9	28.0	7.8
Motor skills	17.8	1–37	18.6	16.9	17.8	24.3	9.3

Data on daily living skills was available for 92 adults. Half of the adults were not toilet trained. Of the remaining adults, half were toilet trained and indicated that they had to go to the toilet, the other half were clockwise trained. Most adults (59%) could eat independently with cutlery (fork and spoon), the other adults needed help with eating, while 47% could drink independently, with or without a no‐spill cup. Parents and/or caregivers of 74 adults did not indicate a decline in these skills with aging. Five adults became incontinent, while they were toilet trained in the past and two adults lost their ability to eat and drink independently.

### 3.11 Communication

Twenty out of 92 adults were able speak some single words and two adults were able to speak short sentences. All remaining adults were completely nonverbal. Gestures, (icon)images and speech generating devices were used by 26%, 28%, and 4%, respectively.

### 3.12 Genotype–phenotype relations

The phenotype in adults with interstitial deletions of the 15q11‐13 region appeared somewhat more severe. Adults from the non‐deletion group achieved significantly higher scores on the Vineland domains socialization, motor skills and daily living skills compared to participants with a deletion of the 15q11‐13 region. On the domains communication, scores were not significantly different between the groups.

Adults with a deletion were diagnosed at a slightly younger age compared to adults in the non‐deletion group: mean age 34 versus 42 months (*p* = .16), which was not significantly different. Significantly more adults in the deletion group suffered from epilepsy. Scoliosis was found in 63% of adults in the deletion group to 45% in the non‐deletion group, but this comparison was also not significant. No differences were found in the severity of epilepsy, height and SDSC scores and the occurrence of constipation, reflux, microcephaly, and sleep problems between the groups (Table 11).

**TABLE 11:** Genotype–phenotype correlations

	Deletion	Non‐deletion	*p*‐value
Age at diagnosis (median age in years)	7	11	.16
Epilepsy (%)	96.4	76.2	.01
Sleep problems (%)	92.6	85.0	.32
Length (<2*SD*) (%)	21.7	31.6	.40
Scoliosis (%)	62.5	45.0	.20
Microcephaly (%)	33.2	25.3	.57
Constipation (%)	87.0	95.0	.33
Reflux (%)	38.9	35.0	.76
VABS scores (mean age in months)			
Communication	12	12	.79
Socialization	10	16	.00
Motor skills	15	24	.00
Daily living skills	16	25	.00

Abbreviation: VABS, Vineland adaptive behavior scales.

### 3.13 Age at death and cause of death

Two adults died during the time frame of our study. One woman died of urosepsis at the age of 83 years. Another woman died of double‐sided and metastatic breast cancer at the age of 49 years.

## 4 DISCUSSION

Here we describe the clinical characteristics of the largest and oldest group of adults with AS reported to date. Clinical findings described represent a combination of questionnaire data, interviews with care givers, review of medical records (including genetic etiology) and physical examination. This is unique for this age group and provides a complete overview of the clinical characteristics of adults with AS.

As expected, our study cohort shows a similar distribution of genetic subtypes as seen in previous large studies (Bindels‐de Heus et al., 2020; Mertz et al., 2013; Williams, Driscoll, & Dagli, 2010). There were no peculiarities in this study group regarding the course of the pregnancy and birth, which is in agreement with prior studies (Clayton‐Smith & Pembrey, 1992; Moncla et al., 1999; A. Smith et al., 1996). Birth weight was approximately 200 g lower compared to the general population, consistent with previous reports (Bindels‐de Heus et al., 2020; Clayton‐Smith, 1992).

The high number of twin pregnancies was striking. In this study, the number of twin births was 6 per 94 deliveries. This is four times higher than the Dutch Twin Register reported and could not be explained by maternal age (Glasner, van Beijsterveldt, Willemsen, & Boomsma, 2013). As far as we know high numbers of twins are not previously described in Angelman or Prader‐Willi patients in literature. We cannot explain this high number other than coincidence, and a larger group of individuals with AS is needed to make further statements.

First signs and symptoms were noticed in the first year after birth in the majority of participants, and mainly consisted of feeding problems. Consistent with less access to advanced genetic testing in the past, diagnosis was made at a median age of 6.8 years. For the younger individuals in this study median age of diagnosis of 2.5 years old, which is comparable to the age of genetic testing in younger children with AS in The Netherlands (Bindels‐de Heus et al., 2020).

Most parents and/or caregivers (72%) mentioned a decrease of the hyperactive behavior that existed in childhood, what corresponds with previous studies (Clarke & Marston, 2000; Clayton‐Smith & Pembrey, 1992; L. A. E. M. Laan et al., 1996; J. C. Smith, 2001). Hyperactivity scores on the ABC‐C were considerably lower compared the children with AS described by Sadhwani and colleagues (Sadhwani et al., 2019). Parents often mentioned this diminished hyperactivity the biggest change upon becoming adult. However, behavior problems did not disappear simultaneously with the decrease in hyperactivity. Parents and/or caregivers of 80 adults reported inappropriate behavior of their ward and for 22 adults this was perceived as problematic. Many parents thought this behavior reflected a communication problem. This notion is supported by findings from Didden et al., who suggested that problematic behavior serves to some extent the goal of rejecting and protesting (Didden et al., 2009). In several adults, parents stated that improving communication using aided communication reduced behavioral problems. Aided communication was used by the minority of adult patients. Although most appropriate communications methods used for adults with AS should be studied further, communication should receive proper attention in all adults with AS.

Epilepsy is one of the most significant health concerns for individuals with AS. In our group 89% had a history of seizures, which is consisted with prior studies, reporting incidences of 80 up to 96% (Buckley et al., 1998; Buntinx et al., 1995; Clayton‐Smith & Pembrey, 1992; Galvan‐Manso, Campistol, Conill, & Sanmarti, 2005; L. A. E. M. Laan et al., 1996; L. A. Laan et al., 1997; A. Smith et al., 1996; Thibert et al., 2009; Valente et al., 2006). The mean age of onset of epilepsy of 3.3 years also corresponds with medical literature (Buntinx et al., 1995; Clayton‐Smith & Pembrey, 1992; Galvan‐Manso et al., 2005; L. A. Laan et al., 1997; Pelc, Boyd, Cheron, & Dan, 2008; Thibert et al., 2009). Five individuals developed epilepsy between 10 and 22 years of age years, which indicates that children without epilepsy still need to be closely monitored till adulthood. Epilepsy negatively influenced the level of functioning.

Several studies have reported a decrease in seizures during late childhood or puberty (Clayton‐Smith & Pembrey, 1992b; Larson et al., 2015; Pelc et al., 2008; Valente et al., 2006). However, Laan et al. observed that 92% of the adults still experienced epileptic seizures (L. A. Laan et al., 1997). In our study, in 54% of the adults with epilepsy, seizures had been present in the past year. Two of them experienced an exacerbation of epilepsy at the age of 19 and in the mid‐twenties. This is consistent with previous studies that suggest that epilepsy becomes milder as with age, but an exacerbation of the epilepsy around the age of 20 years is possible (Buckley et al., 1998; J. C. Smith, 2001; Thibert et al., 2009).

Most frequent seizure types were atypical absences and tonic–clonic seizures. Episodes of non‐convulsive status epilepticus were not reported in adulthood. These episodes are difficult to recognize and it is possible some episodes in adulthood were missed. Notably, the pattern of epilepsy appears to change into adulthood. In a number of adults, prolonged episodes of rhythmic shaking without loss of consciousness had been observed. These episodes could be triggered by menstruation or sudden changes in position, had a duration of several minutes to hours and were very debilitating for some adults. Such episodes have been described in other cohorts as well (Guerrini et al., 1996; Larson et al., 2015). Further study into the etiology, triggers and treatment of these episodes should be performed to optimize care.

Sleeping disturbances improved (or even disappeared) in the majority of adults. However 32% of the adults still showed behavioral sleep disturbance, which rated as a formal sleeping disorder according to SDSC screening scale, while 57% of the caregivers reported sleep problems at the time of the study. The identification of sleeping problems proved to be difficult and caregivers often claimed unaware of the actual amount of sleep. An accurate and valid method to assess sleep in individuals with AS remains to be studied.

Notably some parents mentioned diminished sleep problems soon after their child started living in a care facility, which was objectified by camera observations during the night. This could suggest that a behavioral pattern can evolve in the home environment, which maintains the sleep problems. Conant et al. suggested that epilepsy and use of AED may be related to higher degree of sleep disturbance in these adults (Conant, Thibert, & Thiele, 2009). However, we did not find any relation between sleep problems and epilepsy in our cohort of adults, in accordance with Bindels‐de Heus et al who could not confirm a relationship in Dutch children (Bindels‐de Heus et al., 2020).

Except for three adults, all adults over 40 years showed a decline in mobility. Clayton‐Smith et al. also observed a decline in mobility in all adults over the years (Clayton‐Smith & Laan, 2003). They claimed mobility was affected by weight gain and development of scoliosis (J. C. Smith, 2001). We could not confirm a relation between overweight or obesity and level of mobility. It was in fact remarkable that the four adults who were underweight, were all wheelchair bound outside the home. The degree of severe scoliosis in non‐ambulators was conspicuous. Out of 11 adults with severe scoliosis, six were wheelchair bound outside the house. Scoliosis and the degree of mobility could certainly have a causal relation, however if mobility is affected by scoliosis or whether development of scoliosis is increased by the limitations in mobility, is unknown. Therefore, we plead for early and frequent monitoring of the spine and timely intervention. In the current study, we did not gather longitudinal data on progression of scoliosis in adults. Although scoliosis predominantly develops during growth, it is interesting to investigate if progression of scoliosis occurs in adults.

Caring for adults with AS, mobility should receive appropriate attention. The ability to walk is likely influenced by weight, ataxia, muscle tone, deviations and/or contractures of hips, knees, ankles and feet, tremor and balance problems. More knowledge is needed to influence these factors in order to preserve the ability to walk in adults.

In more than 30% of the adults in our cohort, visual problems were reported. However, vision tests were often not performed in these adults. The number of adults with visual impairment may be underestimated, since the study of Michielleto et al. reported an incidence of 97% of ametropia (Michieletto, Bonanni, & Pensiero, 2011). Moreover, 32% of our adults had strabismus. It is remarkable that out of four adults over sixty, two had developed cataracts. Buckley et al. also mentioned cataract in three adults developed between the 55th and 58th year (Buckley et al., 1998). This is important to monitor. In addition, there are some cases of keratoconus and retinochoroidal atrophy described in literature (Rufa et al., 2003; Sandanam et al., 1997). In our cohort is keratoconus was described in the medical files by an ophthalmologist in three adults. For most adults this was unknown. It is likely to assume that visual problems are underestimated in adults with AS and important that clinicians are aware of the possibility of ophthalmological and visual problems.

Most (89%) of the adults in our cohort suffered from chronic constipation, which is comparable with a previous report (Larson et al., 2015). This percentage of constipation is very high, even though chronic constipation is already of frequent occurrence in adults with intellectual disabilities. In a study in Dutch elderly people with intellectual disabilities, chronic constipation occurred in 43.3% of adults above 50 years of age (Evenhuis, 2014). Although constipation is only mentioned as an associated (20–80%) feature in the Consensus for Diagnostic Criteria, our results emphasize that it is very important to be aware of constipation in adults with AS (Williams et al., 2006).

The mean Body Mass Index of 24 was rather high, but obesity (BMI ≥30) was found in only 9% of our adults, which is lower than the reported prevalence of obesity in the general Dutch elderly population (10%). Obesity has been described particularly in adult women, but this was not confirmed in this study (J. C. Smith, 2001). According to data from CBS (Statistics Netherlands) the number of Dutch with overweight (BMI ≥25) was nearly half of all adults (48%) in 2012, which corresponds with the prevalence of overweight in our cohort of 46% (Centraal Bureau voor de Statistiek, 2013). The percentage of adults who were underweight was higher in our study (9%), compared to the general Dutch population (3%).

Although the adults we studied did not show a higher prevalence of overweight and obesity, almost half of the care givers mentioned difficulties of their child to master themselves with food. Frequently they mentioned diets to preserve a healthy weight. We suspect the care and focus on a healthy diet of these caregivers lead to the reduction of overweight and obesity in our cohort and recommend monitoring of weight and diet and appropriate support in adults with AS to prevent obesity.

Microcephaly is often described as a characteristic of Angelman syndrome. In the Consensus for Diagnostic Criteria microcephaly is a frequent (>80%) clinical feature of Angelman syndrome (Williams et al., 2006). Although median head circumference was below average, only 27 out of 88 adults (30.7%) showed microcephaly. This is relevant in case of diagnosing elderly individuals with intellectual disabilities.

Since mobility tends to decline in adults with AS, we investigated whether there is also decline of cognitive and other functions over age. Overall, such decline was not evident. Parents of four adults reported loss of abilities. Two adults (age 37 and 69 years) lost their urine continence and two adults (age 23 and 42 years) could eat less independent than before. As to the VABS results, by taking genotypic variability into account, we did not observe an evident decline in cognitive functioning. But a larger group, longitudinal design and extensive cognitive testing is necessary to address this further.

Besides the fact that the prognosis of the syndrome is still unknown, there is almost no data on life expectancy. Medical literature describes that people with Angelman syndrome have near normal to normal life expectancy. This assumption is based on sporadic case reports of people above 50 years of age with clinical features of Angelman syndrome (Bjerre et al., 1984; Clayton‐Smith & Laan, 2003; Williams et al., 2010). The oldest individual who had been described in the literature so far, was a 75 year old man (Bjerre et al., 1984). The oldest individuals in our cohort were 63, 69, and 83 years old, and all female.

An important factor that may affect the level of adaptive functioning, severity of epilepsy, and possibly life expectancy is genotype. Although there is a large diversity between adults with Angelman syndrome, which cannot be explained by genotype alone, we confirmed the presence of more severe phenotypes in adults with 15q11‐13 deletions (Moncla et al., 1999; A. Smith et al., 1996; Williams et al., 2006). Adults with a deletion showed lower levels of adaptive functioning on the domains socialization, motor skills and daily living skills and more often suffered from epilepsy.

Interestingly no differences on the communication domain of the VABS was found, while clinically adults with UPD and pathological variants of UBES3A appeared to communicate slightly better. Since almost all adults were however nonverbal, differences were subtle and could apparently not be measured in our study. It would be interesting to investigate means to further characterize the communication in AS adults.

## 5 STRENGTHS AND LIMITATIONS

Main strength of this study is the detailed description of clinical characteristics of AS in adulthood, in the largest cohort of AS adults reported to date. Data gathered are combination of questionnaire data, interviews with care givers, review of medical records and physical examination of almost every individual, which is unique and provides a much more complete picture of health issues in adults with AS. More knowledge on the natural course of AS is essential to evaluate the effects of future therapies. Furthermore, we describe areas of concern for physicians caring for adults with AS, hoping this knowledge will improve their care.

A limitation of the study is mainly the cross‐sectional design. Future studies gathering information on the natural course should preferably assemble longitudinal data.

Our study cohort shows a distribution of genetic subtypes as seen in the general AS population (Williams et al., 2010). Unfortunately, the precise genetic subtype was not available for 18 adults. Besides no adults with an confirmed imprinting center defect were included in our study.

In this study, the VABS was used to estimate the level of adaptive functioning on the domains communication, daily living skills, socialization and motor skills. Although the VABS is widely used and construct validity is presumably high, this tool is only used in children and adolescents and not in the adult population, as for the SDSC that is also studied in children only. Furthermore although considerable differences in communication were observed by the researchers between individuals with AS, this was not reflected in the VABS scores on the domain communication. Further studies into methods to provide more detailed information on functioning on the domain communication of the adults with AS will have to be studied.

Data collected in this study were partly qualitative and open to interpretation. This was especially the case in assessing for example behavioral and sleeping problems. We tried to limit the bias of interpretation through the use of the different questionnaires and researchers discussed the outcomes together.

As expected, we had some missing medical data. Although this could in part be substituted by interviews with care‐takers after the completion of the questionnaires, a large proportion of the data was collected retrospectively, with a risk of recall bias. For nine adults, we did not have consent for physical examination and in a small number of patients, parts of the physical examination could not be completed due to resistance. We were not able to include deceased patients in the study and hence we could not gather reliable data on life expectancy.

## 6 CONCLUSION

This is the most comprehensive survey of health issues in adults with AS, including physical examination and in person interviews with caregivers, reported in literature to date. Adults with AS are relatively healthy, but still have some serious debilitating health problems. Care for adults with AS should focus on monitoring weight, visual functioning and management of epilepsy, behavioral and sleep problems, communication, reflux, and constipation. Frequent monitoring of the spine and timely intervention when scoliosis is developing is important as well as the monitoring of mobility and factors associated with a decline of mobility such as overweight and deviations and/or contractures of joints to prevent deterioration of walking.

To learn more about the course of AS in adulthood, a larger cohort is required. To identify putative adult AS patients, it is important to realize that only the minority of adults with AS have microcephaly. Additional areas of future research include level of cognitive functioning in relation to the age, life expectancy, ophthalmologic pathology, further genotype–phenotype relations, and twin pregnancies. Furthermore, factors associated with the decline in mobility should be studied to prevent the deterioration of walking seen in a large number of the adults with Angelman and features of epilepsy in adulthood should be studied further in combination with the most effective treatment of epilepsy, since epilepsy proved to have a negative association with the level of cognitive functioning and mobility.

Hopefully, the increase in knowledge on the natural course of AS in adulthood will result in an earlier diagnosis and a subsequent improvement in care.

### ENCORE Expertise Center for AS 18+

Karen G. C. B. Bindels‐de Heus, Alice S. Brooks, Hennie T. Brüggenwirth, Leontine W. ten Hoopen, Maartje ten Hooven‐Radstaake, Bianca M. van Iperen‐Kolk, Henriëtte A. Moll, Sabine E. Mous, Cindy Navis, Sandra Titulaer, Marlies J. Valstar, Marie‐Claire Y. de Wit.

## CONFLICT OF INTEREST

The authors declare no potential conflict of interest.

## AUTHOR CONTRIBUTIONS

Inge den Besten, Rianne F. de Jong, Amber Geerts‐Haages, and Marlies J. Valstar visited the patients, conducted interviews and performed clinical examination, studied the medical records and analyzed the clinical data. Hennie T Bruggenwirth performed further genetic analysis and together with Alice Brooks and Marije Koopmans helped with the interpretation and description of the genetic results. Marlies J. Valstar supervised the research and Ype Elgersma and Dederieke A.M. Festen helped supervising the research. Inge den Besten and Marlies J. Valstar wrote the first draft of the manuscript. All authors, including the members of the ENCORE Expertise Center for AS 18+, reviewed the manuscript.

## Data Availability

The data that support the findings of this study are available from the corresponding author upon reasonable request within national legal bounds.

## References

[ajmga61940-bib-0001] Aman, M. G. , Singh, N. N. , Stewart, A. W. , & Field, C. J. (1985). Psychometric characteristics of the aberrant behavior checklist. American Journal of Mental Deficiency, 89(5), 492–502. 3158201

[ajmga61940-bib-0002] Angelman, H. (1965). ‘Puppet’ children a report on three cases. Developmental Medicine & Child Neurology, 7(6), 681–688. 10.1111/j.1469-8749.2008.03035.x 18754889

[ajmga61940-bib-0003] Beygo, J. , Buiting, K. , Ramsden, S. C. , Ellis, R. , Clayton‐Smith, J. , & Kanber, D. (2019). Update of the EMQN/ACGS best practice guidelines for molecular analysis of Prader‐Willi and Angelman syndromes. European Journal of Human Genetics, 27(9), 1326–1340. 10.1038/s41431-019-0435-0 31235867 PMC6777528

[ajmga61940-bib-0004] Bindels‐de Heus, K. , Mous, S. E. , Ten Hooven‐Radstaake, M. , van Iperen‐Kolk, B. M. , Navis, C. , Rietman, A. B. , … de Wit, M. Y. (2020). An overview of health issues and development in a large clinical cohort of children with Angelman syndrome. American Journal of Medical Genetics Part A, 182(1), 53–63. 10.1002/ajmg.a.61382 31729827 PMC6916553

[ajmga61940-bib-0005] Bjerre, I. , Fagher, B. , Ryding, E. , & Rosen, I. (1984). The Angelman or ‘happy puppet’ syndrome. Clinical and electroencephalographic features and cerebral blood flow. Acta Paediatrica Scandinavica, 73(3), 398–402. 6741540 10.1111/j.1651-2227.1994.tb17755.x

[ajmga61940-bib-0006] Brinkley, J. , Nations, L. , Abramson, R. K. , Hall, A. , Wright, H. H. , Gabriels, R. , … Cuccaro, M. L. (2007). Factor analysis of the aberrant behavior checklist in individuals with autism spectrum disorders. Journal of Autism and Developmental Disorders, 37(10), 1949–1959. 10.1007/s10803-006-0327-3 17186368

[ajmga61940-bib-0007] Brown, E. C. , Aman, M. G. , & Havercamp, S. M. (2002). Factor analysis and norms for parent ratings on the aberrant behavior checklist‐community for young people in special education. Research in Developmental Disabilities, 23(1), 45–60. 10.1016/s0891-4222(01)00091-9 12071395

[ajmga61940-bib-0008] Bruni, O. , Ottaviano, S. , Guidetti, V. , Romoli, M. , Innocenzi, M. , Cortesi, F. , & Giannotti, F. (1996). The sleep disturbance scale for children (SDSC). Construction and validation of an instrument to evaluate sleep disturbances in childhood and adolescence. Journal of Sleep Research, 5(4), 251–261. 10.1111/j.1365-2869.1996.00251.x 9065877

[ajmga61940-bib-0009] Buckley, R. H. , Dinno, N. , & Weber, P. (1998). Angelman syndrome: Are the estimates too low? American Journal of Medical Genetics, 80(4), 385–390. 10.1002/(sici)1096-8628(19981204)80:4<385::aid-ajmg15>3.0.co;2-9 9856568

[ajmga61940-bib-0010] Buiting, K. , Williams, C. , & Horsthemke, B. (2016). Angelman syndrome—insights into a rare neurogenetic disorder. Nature Reviews Neurology, 12(10), 584–593. 10.1038/nrneurol.2016.133 27615419

[ajmga61940-bib-0011] Buntinx, I. M. , Hennekam, R. C. , Brouwer, O. F. , Stroink, H. , Beuten, J. , Mangelschots, K. , & Fryns, J. P. (1995). Clinical profile of Angelman syndrome at different ages. American Journal of Medical Genetics, 56(2), 176–183. 7625442 10.1002/ajmg.1320560213

[ajmga61940-bib-0012] Centraal Bureau voor de Statistiek . (2013). Statline. The Hague, The Netherlands: CBS. http://statline.cbs.nl/statweb/.

[ajmga61940-bib-0013] Clarke, D. J. , & Marston, G. (2000). Problem behaviors associated with 15q‐ Angelman syndrome. American Journal of Mental Retardation, 105(1), 25–31. 10683706 10.1352/0895-8017(2000)105<0025:PBAWQA>2.0.CO;2

[ajmga61940-bib-0014] Clayton‐Smith, J. (1993). Clinical research on Angelman syndrome in the United Kingdom: Observations on 82 affected individuals. American Journal of Medical Genetics, 46(1), 12–15. 7684188 10.1002/ajmg.1320460105

[ajmga61940-bib-0015] Clayton‐Smith, J. , & Laan, L. (2003). Angelman syndrome: A review of the clinical and genetic aspects. Journal of Medical Genetics, 40, 87–95. 12566516 10.1136/jmg.40.2.87 PMC1735357

[ajmga61940-bib-0316] Clayton‐Smith, J. (1992). Angelman syndrome. Archives of Disease in Childhood, 67(7), 889–890. 1519953 10.1136/adc.67.7.889 PMC1793843

[ajmga61940-bib-0016] Clayton‐Smith, J. , & Pembrey, M. E. (1992). Angelman syndrome. Journal of Medical Genetics, 29(6), 412–415. 1619637 10.1136/jmg.29.6.412 PMC1015993

[ajmga61940-bib-0017] Conant, K. D. , Thibert, R. L. , & Thiele, E. A. (2009). Epilepsy and the sleep‐wake patterns found in Angelman syndrome. Epilepsia, 50(11), 2497–2500. 19453716 10.1111/j.1528-1167.2009.02109.x

[ajmga61940-bib-0018] de Bildt, A. , Kraijer, D. , Sytema, S. , & Minderaa, R. (2005). The psychometric properties of the Vineland adaptive behavior scales in children and adolescents with mental retardation. Journal of Autism and Developmental Disorders, 35(1), 53–62. 10.1007/s10803-004-1033-7 15796122

[ajmga61940-bib-0019] Didden, R. , Sigafoos, J. , Korzilius, H. , Baas, A. , Lancioni, G. E. , O'Reilly, M. F. , & Curfs, L. M. G. (2009). Form and function of communicative behaviours in individuals with Angelman syndrome. Journal of Applied Research in Intellectual Disabilities, 22, 526–537.

[ajmga61940-bib-0020] Duker, P. C. , Van Driel, S. , & Van De Bercken, J. (2002). Communication profiles of individuals with Down's syndrome, Angelman syndrome and pervasive developmental disorder. Journal of Intellectual Disability Research, 46(1), 35–40. 10.1046/j.1365-2788.2002.00355.x 11851854

[ajmga61940-bib-0021] Evenhuis, H. (2014). Resultaten van de GOUD‐studie 2008–2013. Ned Tijdschr Geneeskd, 158, A8016. 25424629

[ajmga61940-bib-0022] Galvan‐Manso, M. , Campistol, J. , Conill, J. , & Sanmarti, F. X. (2005). Analysis of the characteristics of epilepsy in 37 patients with the molecular diagnosis of Angelman syndrome. Epileptic Disorders, 7(1), 19–25. 15741136

[ajmga61940-bib-0023] Geerts‐Haages, A. , Bossuyt, S. N. V. , den Besten, I. , Bruggenwirth, H. , van der Burgt, I. , Yntema, H. G. , … Valstar, M. (2020). A novel UBE3A sequence variant identified in eight related individuals with neurodevelopmental delay, results in a phenotype which does not match the clinical criteria of Angelman syndrome. Molecular Genetics & Genomic Medicine, 5, e1481 10.1002/mgg3.1481 PMC7667313 32889787

[ajmga61940-bib-0024] Giroud, M. , Daubail, B. , Khayat, N. , Chouchane, M. , Berger, E. , Muzard, E. , … Moulin, T. (2015). Angelman syndrome: A case series assessing neurological issues in adulthood. European Neurology, 73(1–2), 119–125. 25472600 10.1159/000369454

[ajmga61940-bib-0025] Glasner, T. J. , van Beijsterveldt, C. E. M. , Willemsen, G. , & Boomsma, D. I. (2013). Meerlinggeboorten in Nederland. Nederlands Tijdschrift voor Geneeskunde, 157(A5962), 1–5. 23890166

[ajmga61940-bib-0026] Guerrini, R. , De Lorey, T. M. , Bonanni, P. , Monda, A. , Dravet, C. , Suisse, G. , … Serratosa, J. M. (1996). Cortical myoclonus in Angelman syndrome. Annals of Neurology, 40(1), 39–48. 10.1002/ana.410400109 8687190

[ajmga61940-bib-0027] Jacobsen, J. , King, B. H. , Leventhal, B. L. , Christian, S. L. , Ledbetter, D. H. , & Cook, E. H., Jr. (1998). Molecular screening for proximal 15q abnormalities in a mentally retarded population. Journal of Medical Genetics, 35(7), 534–538. 9678696 10.1136/jmg.35.7.534 PMC1051362

[ajmga61940-bib-0028] Laan, L. A. , Renier, W. O. , Arts, W. F. , Buntinx, I. M. , vd Burgt, I. J. , Stroink, H. , … Brouwer, O. F. (1997). Evolution of epilepsy and EEG findings in Angelman syndrome. Epilepsia, 38(2), 195–199. 9048672 10.1111/j.1528-1157.1997.tb01097.x

[ajmga61940-bib-0029] Laan, L. A. E. M. , Den Boer, A. T. , Hennekam, R. C. M. , Renier, W. O. , & Brouwer, O. F. (1996). Angelman syndrome in adulthood. American Journal of Medical Genetics, 66(3), 356–360. 10.1002/(sici)1096-8628(19961218)66:3<356::aid-ajmg21>3.0.co;2-k 9072912

[ajmga61940-bib-0030] Larson, A. M. , Shinnick, J. E. , Shaaya, E. A. , Thiele, E. A. , & Thibert, R. L. (2015). Angelman syndrome in adulthood. American Journal of Medical Genetics Part A, 167(2), 331–344. 10.1002/ajmg.a.36864 PMC5534346 25428759

[ajmga61940-bib-0031] Leitner, R. P. , & Smith, A. (1996). An Angelman syndrome clinic: Report on 24 patients. Journal of Paediatrics and Child Health, 32(2), 94–98. 8860380 10.1111/j.1440-1754.1996.tb00902.x

[ajmga61940-bib-0032] Marshburn, E. C. , & Aman, M. G. (1992). Factor validity and norms for the aberrant behavior checklist in a community sample of children with mental retardation. Journal of Autism and Developmental Disorders, 22(3), 357–373. 1383187 10.1007/BF01048240

[ajmga61940-bib-0033] Meijers‐Heijboer, E. J. , Sandkuijl, L. A. , Brunner, H. G. , Smeets, H. J. , Hoogeboom, A. J. , Deelen, W. H. , … Niermeijer, M. F. (1992). Linkage analysis with chromosome 15q11‐13 markers shows genomic imprinting in familial Angelman syndrome. Journal of Medical Genetics, 29(12), 853–857. 1362220 10.1136/jmg.29.12.853 PMC1016200

[ajmga61940-bib-0034] Mertz, L. G. , Christensen, R. , Vogel, I. , Hertz, J. M. , Nielsen, K. B. , Gronskov, K. , & Ostergaard, J. R. (2013). Angelman syndrome in Denmark. Birth incidence, genetic findings, and age at diagnosis. American Journal of Medical Genetics Part A, 161A(9), 2197–2203. 23913711 10.1002/ajmg.a.36058

[ajmga61940-bib-0035] Mertz, L. G. , Thaulov, P. , Trillingsgaard, A. , Christensen, R. , Vogel, I. , Hertz, J. M. , & Ostergaard, J. R. (2014). Neurodevelopmental outcome in Angelman syndrome: Genotype‐phenotype correlations. Research in Developmental Disabilities, 35(7), 1742–1747. 10.1016/j.ridd.2014.02.018 24656292

[ajmga61940-bib-0036] Michieletto, P. , Bonanni, P. , & Pensiero, S. (2011). Ophthalmic findings in Angelman syndrome. Journal of AAPOS, 15(2), 158–161. 10.1016/j.jaapos.2010.12.013 21596294

[ajmga61940-bib-0037] Minassian, B. A. , DeLorey, T. M. , Olsen, R. W. , Philippart, M. , Bronstein, Y. , Zhang, Q. , … Delgado‐Escueta, A. V. (1998). Angelman syndrome: Correlations between epilepsy phenotypes and genotypes. Annals of Neurology, 43(4), 485–493. 9546330 10.1002/ana.410430412

[ajmga61940-bib-0038] Moncla, A. , Malzac, P. , Livet, M. O. , Voelckel, M. A. , Mancini, J. , Delaroziere, J. C. , … Mattei, J. F. (1999). Angelman syndrome resulting from UBE3A mutations in 14 patients from eight families: Clinical manifestations and genetic counselling. Journal of Medical Genetics, 36(7), 554–560. 10424818 PMC1734398

[ajmga61940-bib-0039] Pelc, K. , Boyd, S. G. , Cheron, G. , & Dan, B. (2008). Epilepsy in Angelman syndrome. Seizure, 17(3), 211–217. 10.1016/j.seizure.2007.08.004 17904873

[ajmga61940-bib-0040] Penner, K. A. , Johnston, J. , Faircloth, B. H. , Irish, P. , & Williams, C. A. (1993). Communication, cognition, and social interaction in the Angelman syndrome. American Journal of Medical Genetics, 46(1), 34–39. 8494032 10.1002/ajmg.1320460108

[ajmga61940-bib-0041] Ronan, A. , Buiting, K. , & Dudding, T. (2008). Atypical Angelman syndrome with macrocephaly due to a familial imprinting center deletion. American Journal of Medical Genetics Part A, 146(1), 78–82. 10.1002/ajmg.a.31952 17975803

[ajmga61940-bib-0042] Rufa, A. , Dotti, M. T. , Orrico, A. , Battisti, C. , Carletto, F. , & Federico, A. (2003). Retinochoroidal atrophy in two adult patients with Angelman syndrome. American Journal of Medical Genetics Part A, 122A(2), 155–158. 12955768 10.1002/ajmg.a.20217

[ajmga61940-bib-0043] Sadhwani, A. , Willen, J. M. , LaVallee, N. , Stepanians, M. , Miller, H. , Peters, S. U. , … Tan, W. H. (2019). Maladaptive behaviors in individuals with Angelman syndrome. American Journal of Medical Genetics Part A, 179(6), 983–992. 10.1002/ajmg.a.61140 30942555 PMC8407596

[ajmga61940-bib-0044] Sandanam, T. , Beange, H. , Robson, L. , Woolnough, H. , Buchholz, T. , & Smith, A. (1997). Manifestations in institutionalised adults with Angelman syndrome due to deletion. American Journal of Medical Genetics, 70(4), 415–420. 10.1002/(sici)1096-8628(19970627)70:4<415::aid-ajmg16>3.0.co;2-k 9182785

[ajmga61940-bib-0045] Smith, A. , Wiles, C. , Haan, E. , McGill, J. , Wallace, G. , Dixon, J. , … Trent, R. J. (1996). Clinical features in 27 patients with Angelman syndrome resulting from DNA deletion. Journal of Medical Genetics, 33(2), 107–112. 8929945 10.1136/jmg.33.2.107 PMC1051834

[ajmga61940-bib-0046] Smith, J. C. (2001). Angelman syndrome: Evolution of the phenotype in adolescents and adults. Developmental Medicine & Child Neurology, 43(7), 476–480. 11463179 10.1017/s0012162201000871

[ajmga61940-bib-0047] Sparrow, S. S. , & Cicchetti, D. V. (1985). Diagnostic uses of the Vineland adaptive behavior scales. Journal of Pediatric Psychology, 10(2), 215–225. 4020603 10.1093/jpepsy/10.2.215

[ajmga61940-bib-0048] Tan, W. H. , Bacino, C. A. , Skinner, S. A. , Anselm, I. , Barbieri‐Welge, R. , Bauer‐Carlin, A. , … Bird, L. M. (2011). Angelman syndrome: Mutations influence features in early childhood. American Journal of Medical Genetics Part A, 155(1), 81–90. 10.1002/ajmg.a.33775 PMC3563320 21204213

[ajmga61940-bib-0049] Thibert, R. L. , Conant, K. D. , Braun, E. K. , Bruno, P. , Said, R. R. , Nespeca, M. P. , & Thiele, E. A. (2009). Epilepsy in Angelman syndrome: A questionnaire‐based assessment of the natural history and current treatment options. Epilepsia, 50(11), 2369–2376. 10.1111/j.1528-1167.2009.02108.x 19453717

[ajmga61940-bib-0050] Thibert, R. L. , Larson, A. M. , Hsieh, D. T. , & Raby, A. R. (2013). Neurologic manifestations of Angelman syndrome. Pediatric Neurology, 48, 271–279. 23498559 10.1016/j.pediatrneurol.2012.09.015

[ajmga61940-bib-0051] Valente, K. D. , Koiffmann, C. P. , Fridman, C. , Varella, M. , Kok, F. , Andrade, J. Q. , … Marques‐Dias, M. J. (2006). Epilepsy in patients with angelman syndrome caused by deletion of the chromosome 15q11‐13. Archives in Neurology, 63(1), 122–128. 10.1001/archneur.63.1.122 16401744

[ajmga61940-bib-0052] Williams, C. A. , Angelman, H. , Clayton‐Smith, J. , Driscoll, D. J. , Hendrickson, J. E. , Knoll, J. H. , … Whidden, E. M. (1995). Angelman syndrome: Consensus for diagnostic criteria. Angelman Syndrome Foundation. American Journal of Medical Genetics, 56(2), 237–238. 10.1002/ajmg.1320560224 7625452

[ajmga61940-bib-0053] Williams, C. A. , Beaudet, A. L. , Clayton‐Smith, J. , Knoll, J. H. , Kyllerman, M. , Laan, L. A. , … Wagstaff, J. (2006). Angelman syndrome 2005: Updated consensus for diagnostic criteria. American Journal of Medical Genetics Part A, 140(5), 413–418. 10.1002/ajmg.a.31074 16470747

[ajmga61940-bib-0054] Williams, C. A. , Driscoll, D. J. , & Dagli, A. I. (2010). Clinical and genetic aspects of Angelman syndrome. Genetics in Medicine, 12(7), 385–395. 10.1097/GIM.0b013e3181def138 20445456

[ajmga61940-bib-0055] Williams, C. A. , & Frias, J. L. (1982). The Angelman (“happy puppet”) syndrome. American Journal of Medical Genetics, 11(4), 453–460. 7091188 10.1002/ajmg.1320110411

[ajmga61940-bib-0056] Williams, C. A. , Gray, B. A. , Hendrickson, J. E. , Stone, J. W. , & Cantu, E. S. (1989). Incidence of 15q deletions in the Angelman syndrome: A survey of twelve affected persons. American Journal of Medical Genetics, 32(3), 339–345. 2786338 10.1002/ajmg.1320320313

